# Insight on the regulation mechanism of the nanochannels in hard and brittle materials induced by sparially shaped femtosecond laser

**DOI:** 10.3389/fchem.2022.973570

**Published:** 2022-08-15

**Authors:** Lin Kai, Caiyi Chen, Yu Lu, Yizhao Meng, Yi Liu, Yang Cheng, Qing Yang, Xun Hou, Feng Chen

**Affiliations:** ^1^ State Key Laboratory for Manufacturing System Engineering and Shaanxi Key Laboratory of Photonics Technology for Information, School of Electronic Science and Engineering, Xi’an Jiaotong University, Xi’an, China; ^2^ School of Mechanical Engineering, Xi’an Jiaotong University, Xi’an, China

**Keywords:** laser fabrication, bessel pulses, nanochannels, high aspect ratio, hard and brittle materials

## Abstract

The efficient fabrication of nanochannels on hard and brittle materials is a difficult task in the field of micro and nano processing. We have realized nanochannel arrays on silica with characteristic scales varying from 50–230 nm using a single femtosecond Bessel beam pulse of 515 nm. By characterizing the surface openings, we found that the characteristic scales of the nanopore openings are inextricably linked to the surface energy deposition effect. We achieved not only three asymmetric channel profiles by adjusting the laser-sample interaction region, but also high aspect ratio nanochannels with characteristic scales about 50 nm and aspect ratios over 100. These results on hard and brittle materials provide a broader platform and application scenarios for smart particle rectifiers, DNA molecular sequencing, biosensors, and nanofluidic devices, which are also more suitable for future practical applications due to their low cost, good durability, and high productivity.

## Introduction

Nature is always a treasure trove of human learning such as the surface of lotus leaves (Yong et al., 2013) and the compound eyes of insects (Deng et al., 2016). In recent years, inspired by smart ion channels in cell membranes (Doyle et al., 1998), many scholars are investigating artificial bionic nanochannels (Zhang et al., 2016). Such artificial nanochannels are usually constructed with substrates and then chemically modified to obtain specific responsive properties for different functions (Cairns-Gibson and Cockroft, 2022). The preparation of artificial nanochannels involves the selection of processing methods and materials. The main methods include electron beam processing (Storm et al., 2003; Kim et al., 2006), focused ion beam processing (Patterson et al., 2008; Yang et al., 2011), electrochemical processing (Ayub et al., 2010; Rutkowska et al., 2015), et al. The nanostructures prepared by the above methods have good surface quality and high processing accuracy. However, there are still some problems that cannot be ignored, such as expensive equipment (Shen et al., 2014) and low processing efficiency (Li et al., 2020). In addition, as far as the materials are concerned, hard and brittle materials such as silica and sapphire have excellent application properties such as high light transmission, corrosion and wear resistance compared to semiconductors and polymers, which makes it very valuable in complex practical application scenarios (Liu et al., 2009; He et al., 2012; Tan et al., 2021). On the other hand, their application advantages turn into processing difficulties in the preparation and manufacturing process, which means that it is more difficult to prepare detailed structures on them through the methods mentioned above (Chen et al., 2021). Compared with these methods, femtosecond laser processing has potential for future industrial fabrication because of its wide application range for materials and the possibility of true 3D processing (Lin and Hong, 2021). Nanochannels are ubiquitous structures that play key roles in both biological systems and artificial materials. The applications mainly include electrochemical sensing and biosensing (Zhu et al., 2021), chip laboratory (Pezzuoli et al., 2019), DNA sequencing (Harrell et al., 2004; Fan et al., 2005), and other scientific frontiers. Nevertheless, the use of femtosecond laser technology to obtain structures with feature sizes fabrication less than 100 nm and aspect ratios higher than 20:1 on hard and brittle materials with high thermal and chemical stability, such as ceramics, sapphire, and glass, has rarely been reported.

In this paper, nanochannel arrays can be achieved on silica processed by the femtosecond Bessel pulse, which can be acquired from femtosecond Gaussian beams through spatially shaping. Most of the previous works about femtosecond Bessel pulse processing (Bhuyan et al., 2010, 2014; Rapp et al., 2016) were involved more in the interior of the sample than the role of the interface between silica and air plays on the laser-tuned nanostructure. Here, we focus on the regulation mechanism of nanochannels, namely the role of surface energy deposition during laser-matter interaction. On this basis we obtain a series of asymmetric shaped nanochannels by adjusting the interaction region between the Bessel beam and the sample. What’s more, we also achieve the preparation of nanochannels with an aspect ratio of more than 100:1 with diameter about 50 nm in the region where the nanochannels are just appeared. With the optimization of optical field modulation methods, nanochannels with more complex shapes and more fine structures will be realized in the future, which will promote the vigorous development of nanotechnology.

## Experiment setup and material

**FIGURE 1:**
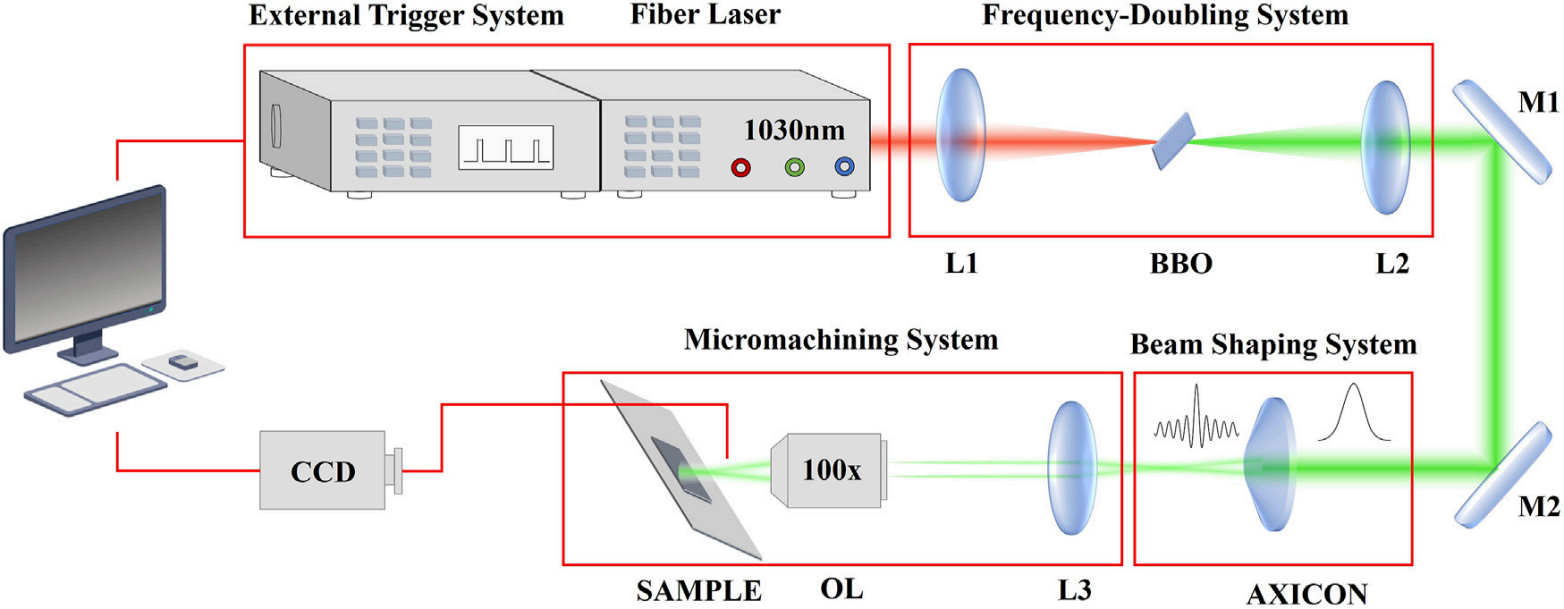
The experimental setup for the preparation of nanostructures on silica. The experimental setup consists of four parts: light source, frequency doubling, beam shaping, and processing stage. L, lens; M, mirror; OL, objective lens.

The experimental system is divided into four parts as Figure 1 shows: fiber laser and external trigger system, frequency-doubling system, beam shaping system and micromachining system. First, we use a femtosecond fiber laser (FemtoYL-20, YSL Photonics Co., Ltd., Wuhan, China) with the central wavelength of 1030 nm and the pulse width of 480 fs, the output repeat frequency of which can be flexible adjusted with the external trigger system. We use the focusing lens to focus the pulsed laser (1030 nm) to the BBO multiplier crystal to output the pulsed laser (515 nm). The diameter of the output laser (515 nm) is 2.0 mm. After that we spatially shape the pulse using an axicon lens (AX251-A, Thorlabs Inc., Newton, MA, United States, 1.0° physical angle) with a base angle of 1° to shape the Gaussian beam into a 0th order Bessel-Gaussian beam with non-diffraction distance and diameter of the core about 10.6 cm and 30.0 μm. The Bessel-Gaussian beam is compressed and projected onto the sample processing area through a micromachining system consisting of lens (160 mm for effective focal length) and an objective lens (×100, 0.8 NA) to achieve preparation on silica (WIN-JGS1-10*10-1, 10*10*1 mm, U-Optic, Wuhan, China) which placed on the piezoelectric translation table (P15.XYZ300S-C1, Resolution: 13 nm, COREMORROW, Inc., Harbin, China).

## Results and discussion

**FIGURE 2:**
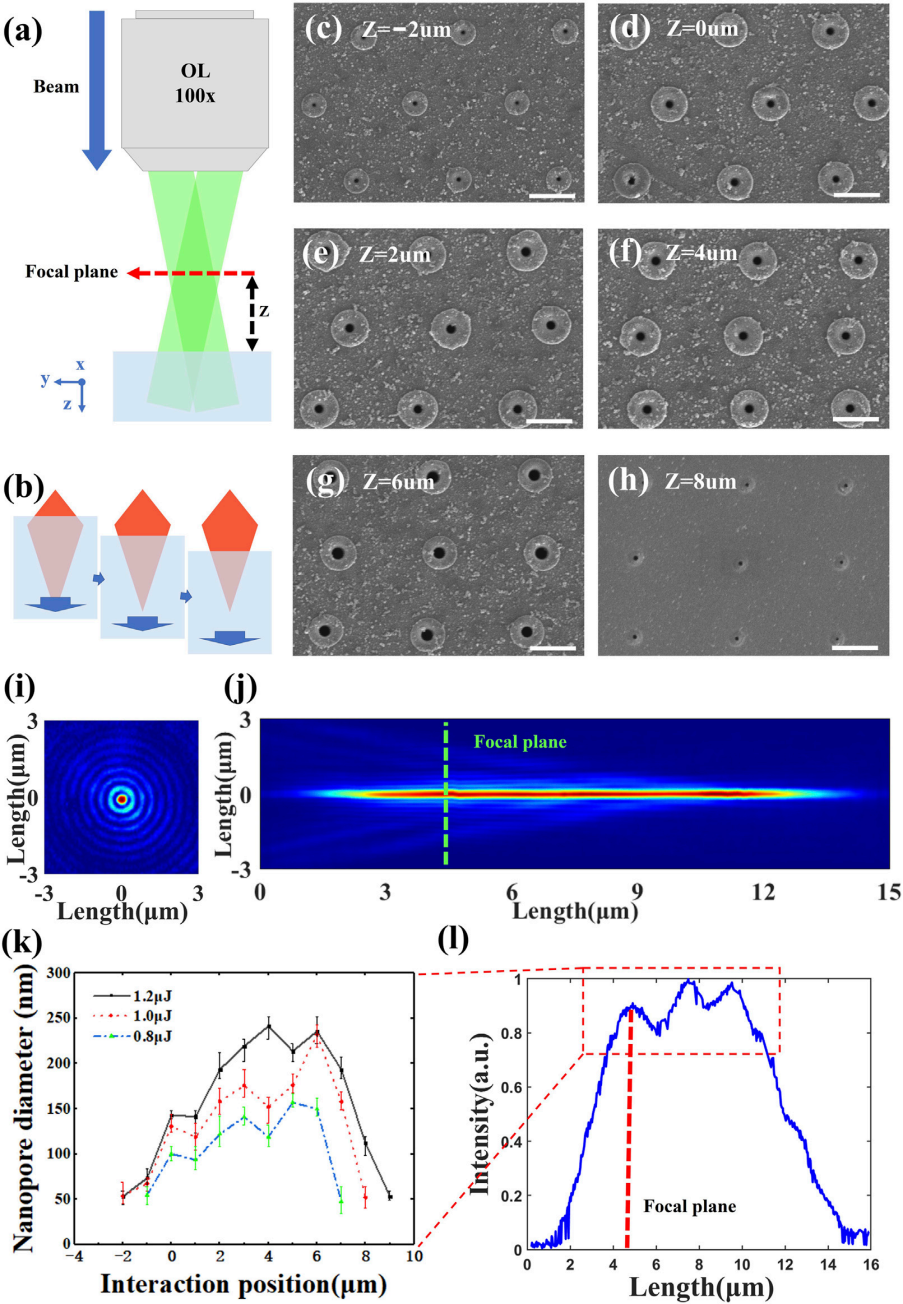
Traversing the region of femtosecond Bessel beam-silica interaction and summarizing the trend of nanopore opening size change due to the interaction region change. **(A)** Schematic diagram of micromachining system. **(B)** Schematic diagram of the variation of the Bessel beam-silica interaction region by the movement of the piezoelectric translation stage. **(C–H)** SEM image of a uniform nanopore array (scale bar 1 μm) with the opening size range corresponding to different interaction regions varying from 50 to 230 nm at a single pulse energy of 1.0 μJ. **(I)** The Bessel beam’s Intensity distribution in the cross-section at focal plane. **(J)** The Bessel beam’s energy distribution along the propagation direction. **(K)** Plot of the nanopore surface opening size with the variation of the interaction region of Bessel beam with silica at single pulse energy 0.8, 1.0, and 1.2 μJ. **(L)** Energy distribution profile of Bessel beam at processing along the propagation direction.

We measured the Bessel beam’s intensity distribution in the cross-section and the energy distribution along the propagation direction with a microscope system. The results are shown in Figures 2I,J. We can see that the energy of Bessel beam is mainly concentrated in the position of the central spot (about 570 nm in diameter and 340 nm in full width half maximum) in the axial propagation. In its direction of propagation, the energy of the Bessel beam within its non-diffraction distance of 13 μm (1–14 μm) is always concentrated for stable transmission at the central spot.

We adjusted the region of interaction of the femtosecond Bessel pulses with the silica in the manner as in Figures 2A,B. As shown in Figure 2A, we define the focal plane of the objective as the reference plane of the laser-sample interaction at Z = 0 μm. We move the Bessel beam up 2 μm to record as “Z = 2 μm,” same below. We used Bessel beam with the pulse energy of 0.8, 1.0, and 1.2 μJ for the preparation of nanochannel arrays spaced at 2 μm, in which every nanochannel is acquired through single femtosecond Bessel pulse procession. The surface morphology measured by the scanning electron microscopy (SEM) are shown in Figures 2C–H, in which the energy of the single femtosecond Bessel pulse is 1.0 μJ. The surface morphology of the nanopore resembles a “volcanic crater” when the focal plane of the objective lens is moved to Z = −2 μm. The opening of the craters gradually becomes larger as Figures 2C–G, as the focal plane moves from Z = −2 μm to Z = 6 μm. Then the opening of the crater rapidly reduces in Figure 2H, as the focal plane moves to Z = 8 μm. The trend of the feature size at the opening of the nanopore with the interaction area of the Bessel beam with the silica for three single-pulse energies is shown in Figure 2K. The energy distribution of the Bessel beam along the propagation direction is shown in Figure 2L. It is very clear that the energy distribution of the Bessel beam along the propagation direction in our processing method corresponds to the energy deposition on the surface of the material during processing. The rapid deposition of energy on the sample surface leads to the rapid removal of material for the removal of the superficial material, which provide room for the inside material removal. The SEM plots show that the results are satisfactory for both the surface opening quality and the uniformity of the nanopores.

**FIGURE 3:**
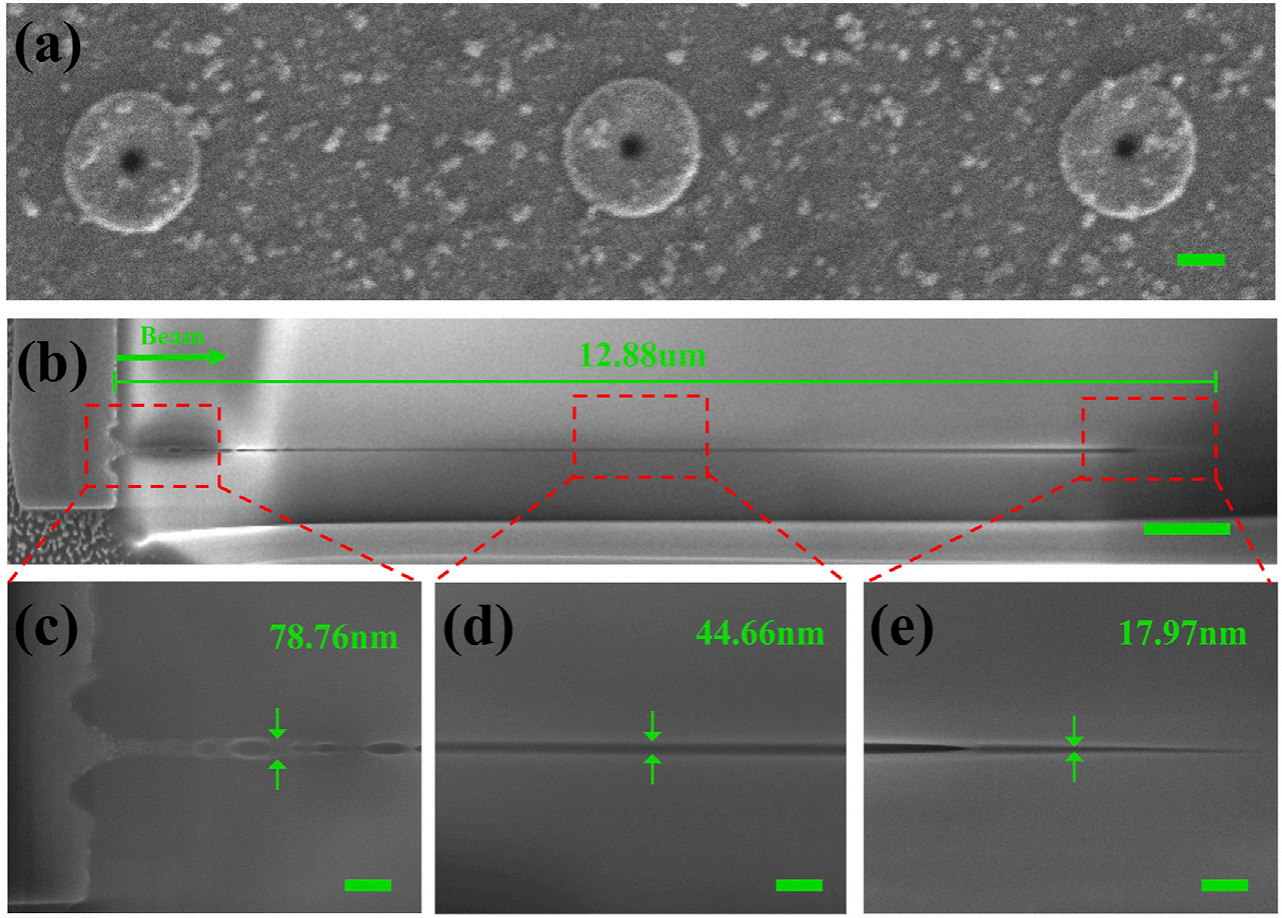
FIB profile results of nanochannel one (surface interaction region located at Z = −1 μm). **(A)** SEM image of nanochannel (scale bar 200 nm). **(B)** FIB results of the overall nanochannel with length about 12.88 μm (scale bar 1 μm). The diameters of the solid-state nanochannel **(C)** head region are ∼80 nm, **(D)** middle region are ∼50 nm, and **(E)** tail region are ∼20 nm (all scale bars 200 nm).

Further, we etch the nanopore array shown in Figure 3A using the focused ion beam (FIB) and observe the cross-sectional situation inside the nanochannel as shown in Figure 3B. A nanochannel with a characteristic scale of about 80 nm and an overall length of 12.88 μm was observed on the surface of the silica sample using a single pulse of Bessel beam with an energy of 1.0 μJ at Z = −1 μm, which has been defined in Figure 2A. The maximum diameter of the head of the nanochannel is about 80 nm. The middle part is a more uniform nanochannel with a diameter of about 50 nm. The tail is a cone-shaped structure with a diameter of about 40 nm as shown in Figures 3C–E. It is notable that the maximum diameter of the nanochannel is less than 100 nm. The length of the nanochannels is 11.2 μm, except for the connected microcavity region (1.6 μm). The average diameter is less than 50 nm. The aspect ratio is about 200:1. Observing the entire channel, a crater-like flared structure forms on the surface of the nanopore’s opening. A series of tiny microcavities are formed below the surface structure as shown in Figure 3C. These cavities are interconnected with each other and become part of the nanochannel. We found that these tiny nanochannels with nanocavities on the top only present when the Bessel beam is like the first case in Figure 2B. According to the previous work (Lu et al., 2021), the plasma below the surface generated by the rapid ionization of electrons absorbing energy significantly hinders the energy deposition in this region, which leads to a decrease in the energy deposition efficiency in the intermediate region compared to the surface and interior. The temperature of the intermediate region is lower than that of the surface and the interior. As a result, the intermediate region is significantly affected by the thermal fluid effect at both ends, resulting in a connected microcavity structure in Figure 3C.

**FIGURE 4:**
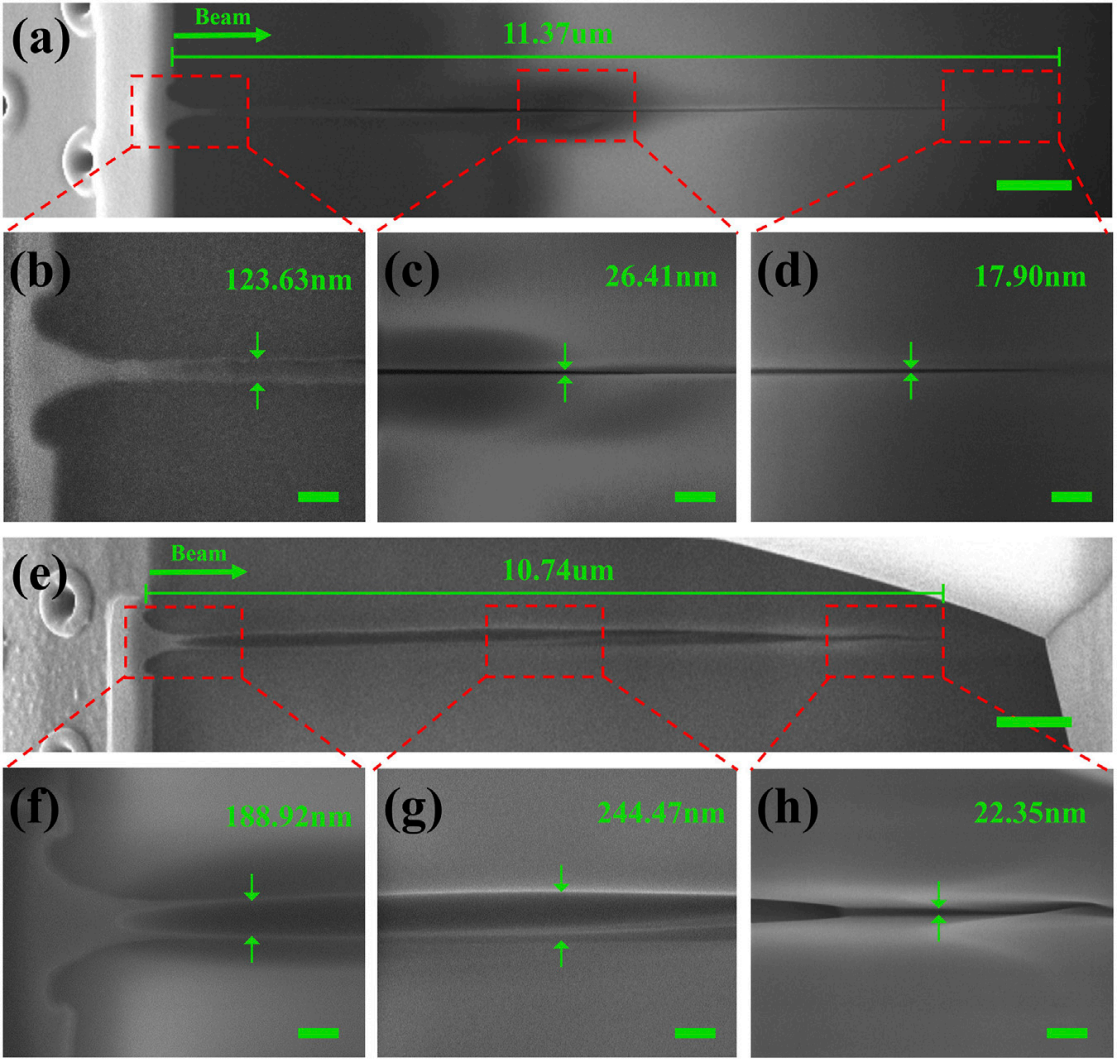
FIB profile results of nanochannel two and three (surface interaction region located at Z = 2 and 5 μm). **(A)** FIB results of the nanochannel two with length about 11.37 μm (scale bar 1 μm). The high-resolution images of the nanochannel two: **(B)** head region, **(C)** middle region, and **(D)** tail region (all scale bars 200 nm). **(E)** FIB results of the nanochannel three with length about 10.74 μm (scale bar 1 μm). The high-resolution images of the nanochannel three: **(F)** head region, **(G)** middle region, and **(H)** tail region (all scale bars 200 nm).

Another two nanopore channels at Z = 2 and 5 μm were also characterized by FIB, as shown in Figures 4A,E. It can be seen that as the sample decreases, the core of the Bessel beam gradually moves away from the interior of the silica sample, like the second and third case in Figure 2B. The morphology of the head region of the nanochannel is no longer a microcavity connecting the two energy deposition regions. Instead, a crater shape exists on the surface, the size of which become increasingly large, as shown in Figures 4B,F. The middle region of the nanochannels loses the binding of the head region, resulting in a more comprehensive material removal, as shown in Figures 4C,G. The area at the end of the nanochannel forms a more uniform conical structure because of the low energy density for uniform deposition as shown in Figures 4D,H. The result of the bending of Figure 4H may be due to the uneven energy deposition caused by the defects or non-uniformity inside the material, which leads to the uneven removal of the material. It can be concluded that the area and energy of the interaction region on the surface of the sample become larger and larger because of the rising Bessel beam, which leads to the removal of more material from the surface of the sample. Consequently, the material in the interaction region becomes more removed, which causes the further increase of the diameter in nanochannel.

**FIGURE 5:**
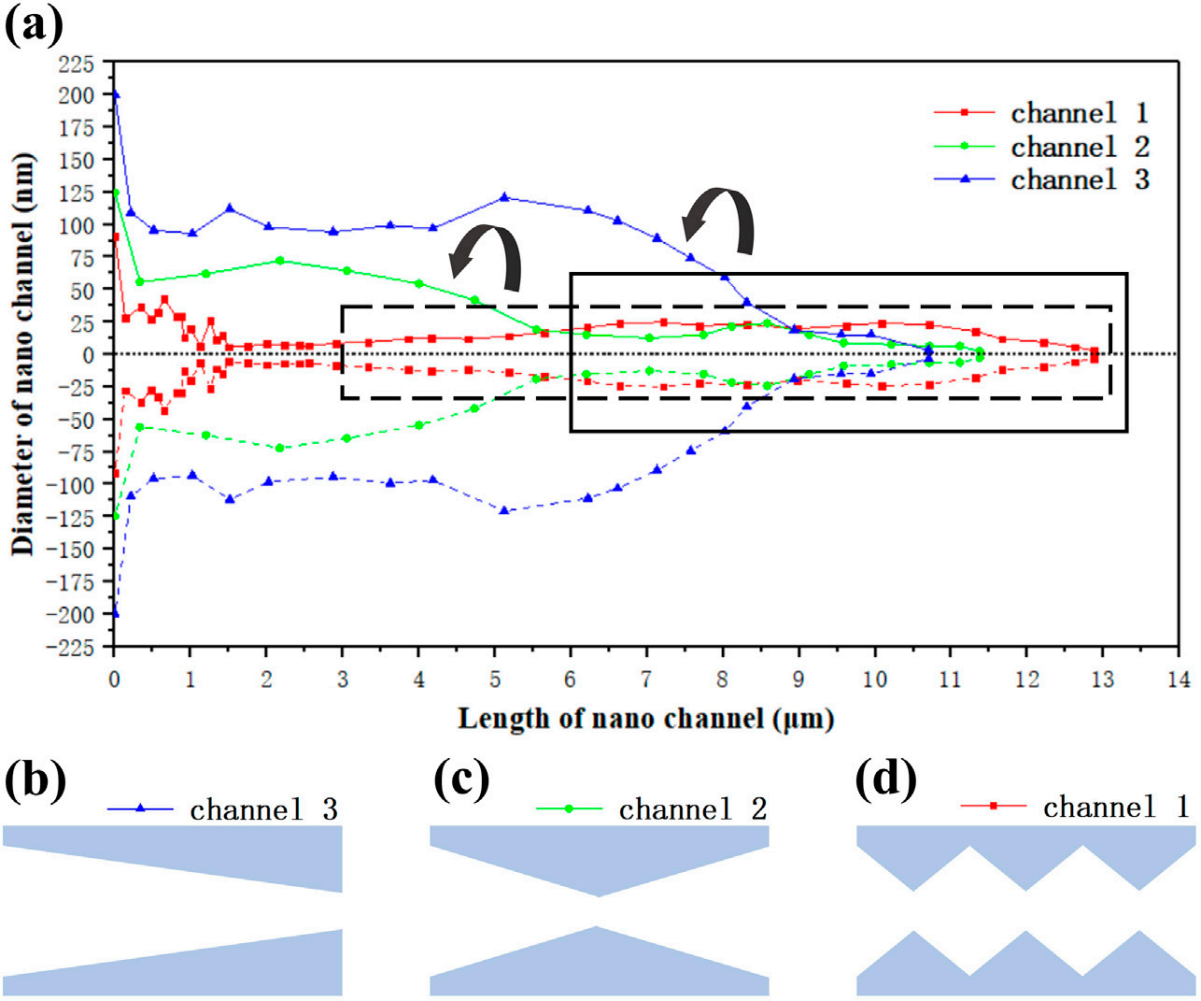
Results of the internal morphological characterization of the nanochannels (color online). **(A)** Channel one (red), channel two (green), and channel three (blue) from inside to outside. The nanochannels of **(B)** type one as conical shape, **(C)** type two as hourglass shape, and **(D)** type three as multi-cavity cascade shape.

For the FIB profiles of the three nanochannels mentioned above, we have characterized their internal diameters. To make the channels more intuitive, we processed the measured diameters into the radius and inverted the internal channel morphology profile symmetrically along the dotted lines as shown in Figure 5A. Form that we can see that the internal contour of channel one can be roughly divided into three cavities, which can be qualitatively attributed to the three peaks of the Bessel pulses used in Figure 2L. The first cavity is about 1.5 μm (0–1.5 μm) in length and 80 nm in the width of maximum. The second cavity is about 4 μm (5–9 μm) in length and 50 nm in the width of maximum. The third cavity is about 2.5 μm (9–11.5 μm) in length and 50 nm in the width of maximum. For channel one, the Bessel beam shares the largest interaction region with the sample causing the longest energy deposition area. Due to the small area of the hot area on the surface, the material removal is limited. Therefore, nanochannels with high aspect ratio appear, as shown in Figure 3B. For channel two, although the Bessel beam slowly moves away from the sample and the laser-sample interaction area decreases, the thermal area of the surface becomes larger and the binding effect on the internal material removal decreases, so the length of the nanochannel increases as shown in Figure 4A. We can see that it has two cavities. The first cavity is about 5.5 μm (0–5.5 μm) in length and 150 nm in the width of maximum. The second cavity is about 2 μm (7.5–9.5 μm) in length and 50 nm in the width of maximum. The curve variation of channel two matches the contour of the dashed part of channel one, as the black arrow shows. For channel three, as the Bessel beam moves further away, the thermal region on the surface becomes larger and gradually approaches the highest energy position, resulting in losing most of the binding of the surface material and the internal material is removed more completely. Which also leads to the channel diameter increases rapidly and is almost connected to the surface opening as shown in Figure 4E. We can be observed to have one cavity. The cavity is about 4.5 μm (4–8.5 μm) in length and 250 nm in the width of maximum. The curve variation of channel three matches the contour of the solid line part of channel one, as the black arrow shows. By summarizing the change in the internal contours above, we can confirm that the area where the Bessel beam interacts with the silica does change by a corresponding distance. We consider the “uncertainty” variation in length (the length of the nanochannels does not match the distance they move) should be explained by the effect of the interaction between the energy deposition on the surface and the energy deposition inside the material during processing.

The interaction between the energy deposition on the surface of the material and the energy deposition inside the material is different due to the different regions where the Bessel beam interacts with the sample, so the morphology of the nanochannels is distinctive. The shape of channel three is similar to a cone as shown in Figure 5B. After appropriating chemical and biological modifications, the channel can be equipped with intelligent biomimetic functions such as light response (LiuDunphy et al., 2004), PH response (Xia et al., 2008), temperature response (Yameen et al., 2009), etc. This asymmetric modification holds potentials in causing a rectifying effect on the particle flow in the channel. The shape of channel two is similar to an hourglass as shown in Figure 5C. Compared with that in Figure 5B, the nanostructures in Figure 5C allows different kinds of chemical and biological modifications on both sides of the hourglass. It can be controlled by two different functions at the same time (Zhang et al., 2013), which is more flexible than a single response channel.The morphology of channel one is like a cascade of multiple cavities like a cell membrane as shown in Figure 5D, which can be regarded as a cascade of hourglasses, which means that each of its narrow port positions can be used as an independent control unit and each control unit can achieve independent variable control, thus achieving more complex gating functions within a single channel.

## Conclusion

In this paper, we have used the single-pulse femtosecond Bessel beam to achieve the preparation of high-speed uniform nanopore arrays on the upper surface of the silica material. When adjusting the interaction area between the Bessel beam and the sample, we noticed that the characteristic size of the nanopore array varies with the energy located on the upper surface of the sample. From which we infer that the size of the direct laser deposition energy on the surface during processing on the upper surface of the silica material has a direct effect on the opening size of the nanopore array. We found that the formation of nanochannels is the result of surface energy deposition and internal energy deposition. The more energy deposited on the surface, the less binding effect on the material below the surface, which will make the material originally insufficient for removal to be removed, thus increasing the overall length of the nanochannel; the energy distribution shape of the beam along the propagation direction will affect the morphology of the nanochannel. In the process of adjusting the relative position of Bessel beam to the sample, we achieved high aspect ratio (more than 100:1) nanochannels on silica with feature size less than 100 nm. Three different nanochannel morphologies were obtained, these asymmetric structures will exhibit gating with polarity after chemical and biological modifications. The more local asymmetry points within a nanochannel, the more variables it can simultaneously possess to regulate. In the future, the combination of spatial light modulator (SLM) and femtosecond laser can achieve more freedom and more flexible optical field shaping in the direction of beam propagation by adjusting the phase of the optical field, which will help us to achieve nanochannels with any desired shape and structure. The preparation of artificial nanochannels with potential for complex bionic functions on hard and brittle substrates that are resistant to corrosion and wear will undoubtedly drive the development of biosensors and nanofluidic devices to more complex areas of chemical and biological environments.

## Data Availability

The original contributions presented in the study are included in the article/supplementary material, further inquiries can be directed to the corresponding authors.
